# Repeated Dietary Exposure to Low Levels of Domoic Acid and Problems with Everyday Memory: Research to Public Health Outreach

**DOI:** 10.3390/toxins10030103

**Published:** 2018-02-28

**Authors:** Lynn M. Grattan, Carol J. Boushey, Yuanyuan Liang, Kathi A. Lefebvre, Laura J. Castellon, Kelsey A. Roberts, Alexandra C. Toben, J. G. Morris

**Affiliations:** 1 Department of Neurology, University of Maryland School of Medicine, Baltimore, MD 21201, USA; lcastellon@som.umaryland.edu (L.J.C.); kelsey.roberts@som.umaryland.edu (K.A.R.); atoben@stetson.edu (A.C.T.); 2 Epidemiology Program, University of Hawaii Cancer Center, Honolulu, HI 96813, USA; cjboushey@cc.hawaii.edu; 3 Department of Epidemiology and Public Health, Division of Biostatistics and Bioinformatics, University of Maryland School of Medicine, Baltimore, MD 21201, USA; yliang@som.umaryland.edu; 4 Environmental and Fisheries Sciences Division, Northwest Fisheries Science Center, National Marine Fisheries Service, National Oceanic and Atmospheric Administration, Seattle, WA 98115, USA; kathi.lefebvre@noaa.gov; 5 Department of Medicine, College of Medicine, and Emerging Pathogens Institute, University of Florida, Gainesville, FL 32608, USA; jgmorris@epi.ufl.edu

**Keywords:** harmful algal blooms, Domoic acid, pseudo-nitzchia, everyday memory, environmental public health, Native American health, Amnesic Shellfish Poisoning, Domoic acid neurotoxicity

## Abstract

Domoic Acid (DA) is a marine-based neurotoxin. Dietary exposure to high levels of DA via shellfish consumption has been associated with Amnesic Shellfish Poisoning, with milder memory decrements found in Native Americans (NAs) with repetitive, lower level exposures. Despite its importance for protective action, the clinical relevance of these milder memory problems remains unknown. The purpose of this study was to determine whether repeated, lower-level exposures to DA impact everyday memory (EM), i.e., the frequency of memory failures in everyday life. A cross-sectional sample of 60 NA men and women from the Pacific NW was studied with measures of dietary exposure to DA via razor clam (RC) consumption and EM. Findings indicated an association between problems with EM and elevated consumption of RCs with low levels of DA throughout the previous week and past year after controlling for age, sex, and education. NAs who eat a lot of RCs with presumably safe levels of DA are at risk for clinically significant memory problems. Public health outreach to minimize repetitive exposures are now in place and were facilitated by the use of community-based participatory research methods, with active involvement of state regulatory agencies, tribe leaders, and local physicians.

## 1. Introduction

There is a growing appreciation of the importance of harmful algal blooms (HABs) and HAB-related illnesses in public health. With dramatic increases in the number of harmful algal blooms as well as the frequency, intensity, and biogeographical expansion of their toxins in coastal regions around the world, the risk of a HAB-related human illnesses is rapidly escalating [1,2]. Health risks are further amplified by increasing rates of fish and seafood consumption in the United States and worldwide [3,4]. Domoic Acid (DA) is a marine-based toxin produced by blooms of some species of the diatom, Pseudo-nitzchia. The toxin is transferred through marine food webs and accumulates in seafood products during harmful algal blooms. 

The potential for serious human illness was first discovered in 1987 when 145 people were sickened after consuming blue mussels contaminated with high levels of DA from Prince Edward Island (PEI), Canada [5,6]. Acute symptoms included vomiting, abdominal cramps, diarrhea, headache, seizures, coma, and in some cases, death [5,6,7]. Among the sentinel cases, several survivors were left with a permanent, severe memory disorder [5,7]. This remarkable symptom, combined with autopsy results indicating significant cell death in the hippocampus, a cerebral region associated with memory, led to the diagnostic label, Amnesic Shellfish Poisoning (ASP). Initially, the toxin responsible for this illness outbreak was unknown. Rapid follow-up work using rodent models led to the identification of DA as the toxin responsible for ASP and to the extrapolation of regulatory safety levels (20 ppm) for human shellfish consumption [8]. Interestingly, DA had been originally discovered in the 1950s in red microalgae, *Chondria armata*, in Japan where it was used medicinally at low doses as an anthelmintic in children with no reported signs of ASP like symptoms [9]. Following the PEI shellfish poisoning event of 1987, toxin monitoring became routine in high risk coastal regions of the United States and Canada. 

Over the past 25 years, measurable levels of DA have been found in the coastal waters and shellfish on the Pacific Coast of the United States. High levels of DA were responsible for outbreaks of toxicity affecting marine birds and sea lions in California, Washington, and Oregon [10,11,12,13]. The most persistent DA producing bloom ever recorded, accompanied by the longest lasting DA levels documented in Pacific razor clams (RC, Siliqua patula) occurred during the 2015–2016 RC season, presumably triggered by warm Pacific Ocean anomalies [14,15]. 

The Pacific RC is a targeted vector for dietary exposure. They depurate DA very slowly and can retain the toxin for up to one year in the natural environment, or several years after being processed, canned, or frozen [16]. To date, effective coastal and shellfish monitoring and well-enforced RC harvesting closures when levels reach 20 ppm have been protective, preventing new cases of ASP. Since the regulatory limits, however, were based upon a single, acute exposure to prevent seizures [17], there may be less dramatic, albeit serious effects associated with repetitive, lower-level exposures. 

Coastal dwelling Native Americans (NAs) in Washington State are at particularly high risk of dietary exposure to DA. They have longstanding historical ties to ancestral coastal waters and beaches and time honored seasonal harvests of the highly prized RC. This traditional food represents more than a protein source in their diet, but is also a central part of their physical, social and spiritual well-being, intergenerational stability, and culture [18,19,20]. Grattan and colleagues [21] studied a cohort (CoASTAL cohort) of NA adults in WA State over four years, documenting DA exposure via RC consumption and memory using standard neuropsychological procedures. They found that NAs who ate more than 15 presumably safe (<20 ppm) RCs per month, over time, suffered mild memory decline, a plausible symptom of DA neurotoxicity. The memory decline could not be explained by age, gender, education, history of substance use, or any acute episodes of ASP. When the study epoch was extended to 8 years, the preliminary findings were replicated, i.e., there was a mild memory decline over 8 years which could not be explained by other factors [18,22,23]. In both studies, the effect size of the memory decrement was small, and overall memory scores remained within normal limits. The findings were consistent with a controlled laboratory study in which weekly exposures to DA at subclinical levels for several weeks resulted in significant learning and memory deficits in mice [24]. While statistically significant and of academic interest, questions remain about the actual clinical significance of the mild memory decrement found in NAs who routinely consume a lot of RCs. Establishing clinical significance is necessary for determinations of the nature and extent of a potential public health problem, and ultimately to provide guidance for HAB management, public health outreach, and community education.

One way to assess the clinical significance of a mild memory decline associated with DA exposure is to examine its functional impacts in daily life. Formal neuropsychological measures of memory, such as the word list recall task used in the previously described studies, have historically been open to criticism regarding their “ecological validity” or their relationship between test performance in the laboratory and forgetfulness in everyday life [25]. An alternative to formal neuropsychological assessments is the measurement of “everyday memory” (EM) or the frequency of memory failures in everyday life [26]. For example, how often does an individual have to recheck whether they have done something they should have done or completely forgotten things they planned to do [25]. 

The purpose of this study was to determine if repeated or cumulative exposure to low levels of DA (<20 ppm) via RC consumption makes an actual difference in EM in the daily lives of coastal dwelling NAs in the Pacific Northwest. Using a cross-sectional study design, 60 NAs were studied with measures of EM and DA exposure (via RC consumption) after a community harvest. It was hypothesized that high RC consumers would have worse EM than non-consumers or low consumers based upon dietary exposure ten days and one year prior to assessment.

## 2. Results

Among the 60 study participants, the median age was 42 years, 56.7% were women and 66.7% had at least high school education (Table 1). The median EMQ-R [25] score for everyday memory was 3 and 51.7% had at least one or more EM problems (a higher EMQ-R score indicates more problems). 

There was no significant difference in age, gender, or education between DA exposure groups for either time epoch (target week or past year). Significant differences in median EMQ-R score were found between the high and low exposure groups for both the target week (4 vs. 1; *p* = 0.003) and past year (4 vs. 1.5; *p* = 0.01). Participants with a high DA exposure were significantly more likely to be in the high every day memory category than those with a low DA exposure (66.7% vs. 27.3%, *p* = 0.01, based on target week DA exposure; 63.3% vs. 26.7%, *p* = 0.01, based on past year DA exposure). When the presence or absence of EM problems were examined, the people in the high exposure group were more likely to report a problem than those in the low exposure group (70.4% vs. 36.4% based on target week exposure; 63.3% vs. 40% based on past year exposure), although the difference was statistically significant for the target week exposure (*p* = 0.02) but not for the past year exposure (*p* = 0.12). 

After adjusting for age, gender, and education (Table 2), DA exposure during target week was not associated with the EMQ-R score. However, the EMQ-R score was significantly associated with the participant’s exposure during the prior year. That is, people with higher level exposures during the past year had more problems with EM (i.e., the total EMQ-R score was about 5-points higher, *p* = 0.04). In addition, there was a non-significant trend towards education being associated with EM. In this case, people with high school education or higher tended to report more problems with EM than those without high school education.

Follow-up studies using logistic regression models indicate that high consumers of RCs were near or more than five times more likely to have more problems with EM (i.e., in the high EM category) than the low consumers (all *p* ≤ 0.005, Table 3). Finally, as Table 4 indicates, when the presence or absence of any memory concern on the EMQ-R was studied, the high consumers were almost four times more likely to report any memory problem than the low consumers during the target week (odds ratio [OR] = 3.92, *p* = 0.03, in the full model; OR = 3.99, *p* = 0.02, in the reduced model). There was a trend for the high consumers during the past year to report a memory problem (OR = 2.52, *p* = 0.11, in the full model; OR = 2.59, *p* = 0.10, in the reduced model), although not statistically significant. There was also a trend for people with higher educational levels to be near or more than three times more likely to report at least one memory problem compared to those with less education (all *p* ≤ 0.10, Table 4).

## 3. Discussion

Overall, the results identified an increase in daily forgetfulness in participants who consumed more RCs (with presumably safe levels of DA) over the past year. High RC consumers (target week, past year) were five to six times more likely to have an elevated EMQ-R score, which could not be explained by age, low education, or sex. High target week RC consumers were more likely to have at least one memory complaint compared to high RC consumers exposed over the past year, but otherwise did not distinguish themselves from the longer exposure duration group. The hypothesis that highly exposed RC consumers would have more EM problems than non-consumers or low consumers over the past year was consistently supported through linear regression and logistic regression models. The hypothesis that high levels of DA exposure during the target week, was partially supported as exposure only predicted EM performance when the outcome measure (EM) was examined categorically. 

The increased likelihood of high RC consumers during target week to report any EM complaints (compared to those exposed during the past year) was unanticipated. Follow-up analyses indicated that this finding could not be explained by the ability of participants to move between the exposure groups. This suggests that time since DA exposure may be an important variable in the chain of events linking RC consumption and exposure to memory outcome. Within the context of the other findings, it also raises the possibility that some EM problems arise and persist for up to 10 to 14 days after exposure, then abate, though never fully to baseline level. The potential for reversibility of DA-related memory deficits in mice was previously demonstrated by Lefebvre et al. [24]. Additionally, previously reported data referencing non-human primate and human models by Kumar et al. [27], suggests a tolerable daily intake (TDI) of 0.075 ppm for gastrointestinal (GI) disturbances in humans. Although our data did not focus on GI disturbances, high RC consumers who exceed this estimated TDI on a regular basis, may also be at risk for more persistent EM problems than those who only consume RCs episodically (such as during the celebrations 10 to 14 days prior to assessment).

There was also an unexpected trend for participants with higher levels of education (high school or greater) to report more EM problems. Since the assessment of EM is based upon self-report, it also includes the process of metamemory, i.e., the ability to have knowledge of one’s own memory capacity and limitations [28]. Metamemory has been previously found to be more accurate in individuals with higher levels of education in healthy aging and dementia populations [28]. 

Everyday memory refers to how memory functions are applied in day to day, real life situations. People with difficulties in EM have to frequently check whether or not they completed a task; forgot to tell someone something important; or failed to remember something they were told the previous day. The overall findings of this study indicate that NAs who consume a lot of RCs have an increased risk for problems with EM, which is functionally relevant and clinically significant. The potential link between EM and DA exposure (target week, past year) is further supported by studies that have found EM to be dependent upon the integrity of the hippocampus and the temporal lobes, cerebral structures known to be disrupted by acute DA exposure and Amnesic Shellfish Poisoning [29,30]. 

In the absence of a reliable human biomarker of DA, all human health studies of DA neurotoxicity to date have relied upon measures of dietary exposure to the toxin. Identification of a reliable human biomarker represents an important direction for future research. An additional limitation of this study is that alcohol and drug use were not factored into the analytic analyses. These were not studied because previous studies of our NA cohort documented minimal alcohol or drug abuse, and neither were contributory to memory problems [21,31]. Finally, questions also remain regarding whether or not the findings from this study of NAs can be generalized to non-NA people in the Pacific Northwest or other coastal regions of the world. It is anticipated that these findings may provide some guidance for future research within the existing cohort and other cohorts in endemic regions.

## 4. Conclusions 

Taking into consideration both the strengths and limitations of this study, combined with the cumulative knowledge of the impacts of consuming high numbers of RCs with relatively low levels of DA, preliminary guidance for DA HAB management as well as public health outreach has been initiated. At this time, the WA Department of Fish and Wildlife has implemented a Razor Clam Advisory [32] encouraging residents to limit RC consumption to fewer than 15 clams/month throughout the course of a year. In addition, pregnant women and the elderly are advised not to eat RCs at all until further information can be obtained specific to these vulnerable groups. At this time, neighboring states are considering adoption of the same advisory. Meanwhile, local physician education is underway and tribe leaders are actively involved in communicating this protective message to tribe members. The ability to seamlessly share these protective messages with tribe leaders and members was made possible because all of the DA studies in the Pacific Northwest to date were based on community-based participatory models.

## 5. Materials and Methods 

### 5.1. Participants

The sample included 60 NA men and women (ages 18–79) from the southern CoASTAL cohort study region in the state of Washington. They represent a volunteer subset of the randomly recruited members of the CoASTAL cohort [31]. Exclusionary criteria for this study included individuals with a history of head injury, dementia, other environmental exposure, or any neurological, psychiatric or medical condition that would potentially impact memory or cognition. The University of Maryland Institutional Review Board approval was obtained prior to consenting participants for this study.

### 5.2. Measures/Procedures

Demographic (age, gender, race, education, and occupational status), medical history, and exposure data were collected using a standard form (modified Boston Occupational and Environmental Neurology Questionnaire). The Everyday Memory Questionnaire-revised (EMQ-R [23]) was used to assess everyday memory. This thirteen-item questionnaire is a self-report measure of memory failures in everyday life. It includes items such as “having to check whether or not you have done something”, “forgetting that you were told something yesterday”, “or forgetting to tell somebody something important”. Each response of the thirteen-item, Likert type response scale will provide a score of 0–5 ranging from “This has not happened” to “this happened five or more times”. The time of reference was established as time since their last RC meal as described below. The total score was based upon the sum of the responses to each item of the measure (possible range 0–65). In this paper, three outcome measures were used: (1) total everyday memory (EM) score; (2) a 2-level EM category (High vs. Low) based on a median split of total EM scores (>3 vs. ≤3); and (3) presence of EM problems based on total EM score (≥1 vs. none).

### 5.3. Dietary Exposure

The Shellfish Assessment Survey (SAS) [33] was administered to participants to examine dietary exposure to DA through RC consumption. As described in Fialkowski et al. [33], the number of clams consumed, as well as the source beaches for harvesting, were identified via the SAS. The time epoch for reporting was the prior 10 days (+/− four days; target week) and the past year. They were all examined over a three-day period two to three weeks after a large community RC harvest with an associated increase in consumption. Typical estimates of DA in RCs from harvesting beaches ranged from 8 to 14 ppm during the study period. These estimates were obtained from the Washington State Department of Health (WDOH) biotoxin monitoring program, which regulates RC harvests based on DA levels detected in RCs via high performance liquid chromatography (HPLC) methods [34]. Typical weight of clam meat consumed was 1.6 ounces (45 g) per clam. Based upon the preferred method for preparation and serving clam chowder in the community under study, a six to eight ounce cup of clam chowder has about 45 g of RC meat and a ten to twelve ounce bowl has 72 g. For data analysis, high and low dietary exposure was based upon a median split of grams consumed during the target week and past year, separately. For data analysis, participants were able to move between different consumption categories for the target week and one year. 

All assessments were conducted using standard procedures by trained examiners (with well-established inter-rater reliability) in field offices on reservation. Participants received $35 remuneration for participation in this study.

### 5.4. Statistical Analysis

Summary statistics were used to describe participants’ characteristics and the differences between the high and low DA exposure groups were compared using Mann–Whitney U test for continuous variables and Chi-square test or Fisher’s exact test for categorical variables. The association between DA exposure and EM measure was examined using linear regression for the continuous outcome (i.e., total EM score) and logistic regression for the dichotomized outcomes, while controlling for potential confounding variables (age, gender, and education). A backward model selection procedure was used so that confounding variables with a *p* value > 0.1 were excluded from the final reduced model. All analyses were performed using Stata/SE (version 15). 

## Figures and Tables

**Table 1:** Participant characteristics by domoic acid exposure group.

Variables	Entire Sample (*n* = 60)	Exposure: Target Week			Exposure: Past Year		
		High (*n* = 27)	Low (*n* = 33)	*p* Value	High (*n* = 30)	Low (*n* = 30)	*p* Value
**Demographics**							
**Age**, year				0.17 ^a^			0.31 ^a^
Median [Q1, Q2]	42 [30, 55.25]	38 [29, 55]	49 [30, 56]		41 [30.25, 54.75]	44 [30, 55.75]	
**Gender**, n(%)				0.68 ^b^			0.79 ^b^
Female	34 (56.67)	14 (51.85)	20 (60.61)		16 (53.33)	18 (60)	
Male	26 (43.33)	13 (48.15)	13 (39.39)		14 (46.67)	12 (40)	
**Education**, n(%)				0.71 ^c^			0.37 ^c^
>/=High School	40 (66.67)	18 (66.67)	22 (66.67)		20 (66.67)	20 (66.67)	
<High School	15 (25)	6 (22.22)	9 (27.27)		6 (20)	9 (30)	
Missing	5 (8.33)	3 (11.11)	2 (6.06)		4 (13.33)	1 (3.33)	
**Outcome measures**							
**EM (Everyday Memory) Score**				0.003 ^a,^*			0.01 ^a,^*
Median [Q1, Q2]	3 [0, 8]	4 [3, 9.5]	1 [0, 4]		4 [3, 10.25]	1.5 [0, 3.75]	
**EM Score Median Split**, n(%)				0.01 ^b,^*			0.01 ^b,^*
High	27 (45)	18 (66.67)	9 (27.27)		19 (63.33)	8 (26.67)	
Low	33 (55)	9 (33.33)	24 (72.73)		11 (36.67)	22 (73.33)	
**EM Problems**, n(%)				0.02 ^b,^*			0.12 ^b^
1 or more	31 (51.67)	19 (70.37)	12 (36.36)		19 (63.33)	12 (40)	
None	29 (48.33)	8 (29.63)	21 (63.64)		11 (36.67)	18 (60)	

^a^ Mann–Whitney U test, ^b^ Chi-squared test, ^c^ Fisher’s exact test; * *p* value < 0.05.

**Table 2:** Adjusted association between Domoic Acid (DA) exposure (measured by target week and annual consumption, respectively) and EM score (continuous) adjusting for age, gender and education using linear regression.

Variable	Full Model			Reduced Model		
	Coefficient	95% CI	*p* Value	Coefficient	95% CI	*p* Value
**DA Exposure: Target Week**						
High vs. Low	1.27	[−3.63, 6.17]	0.60	1.09	[−3.66, 5.85]	0.65
**Age**						
Per 1-year increase	0.05	[−0.11, 0.20]	0.53	-		
**Gender**						
Female vs. Male	−0.99	[−5.97, 3.99]	0.69	-		
**Education**						
>/=H.S. vs. <H.S	4.90	[−0.60, 10.40]	0.08	5.10	[−0.19, 10.40]	0.06
**DA Exposure**: **Past Year**						
High vs. Low	4.94	[0.28, 9.60]	0.04 *	4.70	[0.13, 9.26]	0.04 *
**Age**						
Per 1-year increase	0.06	[−0.08, 0.21]	0.40	-		
**Gender**						
Female vs. Male	−1.04	[−5.81, 3.73]	0.66	-	[−0.43, 9.81]	0.07
**Education**						
>/=H.S vs. <H.S.	4.39	[−0.91, 9.68]	0.10	4.69		

* *p* < 0.05.

**Table 3:** Adjusted association between DA exposure (target week and annual consumption, respectively) and odds of having a high EM score adjusting for age, gender and education using logistic regression.

Variable	Full Model			Reduced Model		
	Odds Ratio	95% CI	*p* Value	Odds Ratio	95% CI	*p* Value
**DA Exposure: Target Week**						
High vs. Low	5.75	[1.70, 19.50]	0.005 *	5.33		
**Age**						
Per 1-year increase	1.02	[0.98, 1.06]	0.29	-	[1.76, 16.14]	0.003 *
**Gender**						
Female vs. Male	1.29	[0.38, 4.32]	0.68	-		
**Education**						
>/=H.S. vs. <H.S	1.11	[0.29, 4.16]	0.88	-		
**DA Exposure: Past Year**						
High vs. Low	5.5	[1.66, 18.22]	0.005 *	4.75		
**Age**						
Per 1-year increase	1.02	[0.98, 1.06]	0.33			0.005 *
**Gender**						
Female vs. Male	1.16	[0.35, 3.87]	0.81		[1.58, 14.25]	
**Education**						
>/=H.S. vs. <H.S	1.03	[0.27, 3.90]	0.97			

* *p* < 0.05.

**Table 4:** Association between DA exposure (target week or past year) and odds of reporting any EMQ-R problem adjusting for age, gender and education.

Variable	Full Model			Reduced Model		
	Odds Ratio	95% CI	*p* Value	Odds Ratio	95% CI	*p* Value
**DA Exposure: Target Week**						
High Consumption	3.92	[1.19, 12.96]	0.03 *	3.99	[1.23, 13.01]	0.02 *
**Age**						
Per 1-year increase	1.00	[0.96, 1.03]	0.84	-		
**Gender**						
Female vs. Male	1.01	[0.31, 3.32]	0.99			
**Education**						
>/=H.S. vs. <H.S	3.26	[0.83, 12.78]	0.09	3.17	[0.84, 11.91]	0.09
**DA Exposure: Past Year**						
High vs. Low	2.52	[0.81, 7.82]	0.11	2.59	[0.84, 7.96]	0.10
**Age**						
Per 1-year increase	0.99	[0.95, 1.03]	0.72	-	-	-
**Gender**						
Female vs. Male	0.94	[0.29, 3.00]	0.91	-	-	-
**Education**						
>/=H.S. vs. <H.S	3.06	[0.82, 11.47]	0.10	2.89	[0.80, 10.37]	0.10

CI: confidence interval; * *p* < 0.05.

## References

[1] Glibert P.M., Anderson D.M., Gentien P., Graneli E., Sellner K.G. (2005). The global complex phenomena of harmful algal blooms. Oceanography.

[2] Grattan L.M., Holobaugh S., Morris J.G. (2016). Harmful algal blooms and public health. Harmful Algae.

[3] Grattan L.M., Holobaugh S., Morris J.G., Morris J.G., Potter M.E. (2013). Seafood Intoxications: Chapter 31. Foodborne Infections and Intoxications.

[4] Van Vorhees D. (2015). Fisheries of the United States 2015: Current Fishery Statistics No. https://www.st.nmfs.noaa.gov/Assets/commercial/fus/fus15/documents/FUS2015.pdf.

[5] Perl T.M., Bédard L., Kosatsky T., Hockin J.C., Todd E.C., Remis R.S. (1990). An outbreak of toxic encephalopathy caused by eating mussels contaminated with domoic acid. N. Engl. J. Med..

[6] Teitelbaum J.S., Zatorre R.J., Carpenter S., Gendron D., Evans A.C., Gjedde A., Cashman N.R. (1990). Neurologic sequelae of domoic acid intoxication due to the ingestion of contaminated mussels. N. Engl. J. Med..

[7] Perl T.M., Bedard L., Kosatsky T., Hockin J.C., Todd E.C., McNutt L.A., Remis R.S. (1990). Amnesic shellfish poisoning: A new clinical syndrome due to domoic acid. Can. Dis. Wkly. Rep..

[8] Wekell J.C., Hurst J., Lefebvre K.A. (2004). The origin of the regulatory limits for PSP and ASP toxins in shellfish. J. Shellfish Res..

[9] Daigo K. (1959). Studies on the constituents of *Chondria armata*, I. Detection of the Anthelmintical Constituents. Yakugaku Zasshi.

[10] Cook P.F., Reichmuth C., Rouse A.A., Libby L.A., Dennison S.E., Carmichael O.T., Kruse-Elliott K.T., Bloom J., Singh B., Fravel V.A. (2015). Algal toxin impairs sea lion memory and hippocampal connectivity, with implications for strandings. Science.

[11] Goldstein T., Mazet J.A., Zabka T.S., Langlois G., Colegrove K.M., Silver M., Bargu S., Van Dolah F., Leighfield T., Conrad P.A. (2008). Novel symptomatology and changing epidemiology of domoic acid toxicosis in California sea lions (*Zalophus californianus*): An increasing risk to marine mammal health. Proc. R. Soc. B.

[12] Wright J.L. (1998). Domoic acid-ten years after. Nat. Toxins.

[13] Scholin C.A., Gulland F., Doucette G.J., Benson S., Busman M., Chavez F.P., Cordaro J., DeLong R., De Vogelaere R., Harvey J. (2000). Mortality of sea lions along the central California coast linked to a toxic diatom bloom. Nature.

[14] McCabe R.M., Hickey B.M., Kudela R.M., Lefebvre K.A., Adams N.G., Bill B.D., Gulland F.M., Thomson R.E., Cochlan W.P., Trainer V.L. (2016). An unprecedented coastwide toxic algal bloom linked to anomalous ocean conditions. Geophys. Res. Lett..

[15] McKibben S.M., Peterson W., Wood M., Trainer V.L., Hunter M., White A.E. (2017). Climatic regulation of the neurotoxin domoic acid. Proc. Natl. Acad. Sci. USA.

[16] Wekell J.C., Gauglitz E.J., Barnett H.J., Hatfield C.L., Simons D., Ayres D. (1994). Occurrence of domoic acid in Washington state razor clams (Siliqua patula) during 1991–1993. Nat. Toxins.

[17] Marien K. (1996). Establishing tolerable Dungeness crab (Cancer magister) and razor clam (Siliqua patula) domoic acid contaminant levels. Environ. Health Perspect..

[18] Grattan L.M., Schumacker J., Reich A., Holobaugh S. Epidemiology and Public Health, Ch 6 Management of Harmful Algal Blooms, 2018.

[19] Reddy S.N. (2015). Feeding family and ancestors: Persistence of traditional Native American lifeways during the Mission Period in coastal Southern California. J. Anthropol. Archaeol..

[20] Schuster R.C., Wein E.E., Dickson C., Chan H.M. (2011). Importance of traditional foods for the food security of two First Nations communities in the Yukon, Canada. Int. J. Circumpolar Health.

[21] Grattan L.M., Boushey C.J., Tracy K., Trainer V.L., Roberts S.M., Schluterman N., Morris J.G. (2016). The association between razor clam consumption and memory in the CoASTAL Cohort. Harmful Algae.

[22] Grattan L.M. Chronic, low-level domoic acid exposure in humans: The CoASTAL cohort. Proceedings of the Domoic Acid Workshop.

[23] Grattan L.M. Impacts of chronic exposure to low levels of domoic acid in Native Americans. Proceedings of the Gordon Research Conference: Mycotoxins and Phycotoxins.

[24] Lefebvre K.A., Kendrick P.S., Ladiges W., Hiolski E.M., Ferriss B.E., Smith D.R., Marcinek D. (2017). Chronic low level DA exposure to the common seafood toxin domoic acid causes cognitive deficits in mice. Harmful Algae.

[25] Royle J., Lincoln N.B. (2008). The everyday memory questionnaire-revised: Development of a 13-item scale. Disabil. Rehabil..

[26] Garcia M.P., Garcia J.F., Guerrero N.V., Triguero J.A., Puente A.E. (1998). Neuropsychological evaluation of everyday memory. Neuropsychol. Rev..

[27] Kumar K.P., Kumar K.S., Nair A.G. (2009). Risk assessment of the amnesic shellfish poison, domoic acid, on animals and humans. J. Environ. Biol..

[28] Szajer J., Murphy C. (2013). Education level predicts retrospective metamemory accuracy in healthy aging and Alzheimer’s disease. J. Clin. Exp. Neuropsychol..

[29] Bjornebekk A., Westlye L.T., Walhovd K.B., Fjell A.M. (2010). Everyday memory: Self-perception and structural brain correlates in a healthy elderly population. J. Int. Neuropsychol. Soc..

[30] Sunderland A., Walker C.M., Walker M.F. (2008). Action errors and dressing disability after stroke: An ecological approach to neuropsychological assessment and intervention. Neuropsychol. Rehabil..

[31] Tracy K., Boushey C.J., Roberts S.M., Morris J.G., Grattan L.M. (2016). Communities advancing the studies of Tribal nations across the lifespan: Design, methods, and baseline of the CoASTAL cohort study. Harmful Algae.

[32] Washington Department State of Health (DOH) Domoic Acid in Razor Clams (DOH No. 332–169). http://www.doh.wa.gov/Portals/1/Documents/Pubs/332-169.pdf.

[33] Fialkowski M.K., McCrory M.A., Roberts S.M., Tracy K., Grattan L.M., Boushey C.J. (2010). Evaluation of dietary assessment tools used to assess the diet of adults participating in the communities advancing the studies of tribal nations across the lifespan cohort. J. Am. Diet. Assoc..

[34] Quilliam M.A., Xie M., Hardstaff W.R. (1991). A Rapid Extraction and Clean-Up Procedure for the Determination of Domoic Acid in Tissue Samples.

